# Imaging of Zebrafish *In Vivo* with Second-Harmonic Generation Reveals Shortened Sarcomeres Associated with Myopathy Induced by Statin

**DOI:** 10.1371/journal.pone.0024764

**Published:** 2011-09-23

**Authors:** Shih-Hao Huang, Chung-Der Hsiao, Dar-Shong Lin, Cho-Yen Chow, Chia-Jen Chang, Ian Liau

**Affiliations:** 1 Department of Applied Chemistry, Institute of Molecular Science, National Chiao Tung University, Hsinchu, Taiwan; 2 Department of Bioscience Technology and Center for Nanotechnology, Chung Yuan Christian University, Chung-Li, Taiwan; 3 Mackay Memorial Hospital, Taipei, Taiwan; Harvard University, United States of America

## Abstract

We employed second-harmonic generation (SHG) imaging and the zebrafish model to investigate the myopathy caused by statin *in vivo* with emphasis on the altered microstructures of the muscle sarcomere, the fundamental contractile element of muscles. This approach derives an advantage of SHG imaging to observe the striated skeletal muscle of living zebrafish based on signals produced mainly from the thick myosin filament of sarcomeres without employing exogenous labels, and eliminates concern about the distortion of muscle structures caused by sample preparation in conventional histological examination. The treatment with statin caused a significantly shortened sarcomere relative to an untreated control (1.73±0.09 µm vs 1.91±0.08 µm, *P*<0.05) while the morphological integrity of the muscle fibers remained largely intact. Mechanistic tests indicated that this microstructural disorder was associated with the biosynthetic pathway of cholesterol, or, specifically, with the impaired production of mevalonate by statins. This microstructural disorder exhibited a strong dependence on both the dosage and the duration of treatment, indicating a possibility to assess the severity of muscle injury according to the altered length of the sarcomeres. In contrast to a conventional assessment of muscle injury using clinical biomarkers in blood, such as creatine kinase that is released from only disrupted myocytes, the ability to determine microstructural modification of sarcomeres allows diagnosis of muscle injury before an onset of conventional clinical symptoms. In light of the increasing prevalence of the incidence of muscle injuries caused by new therapies, our work consolidates the combined use of the zebrafish and SHG imaging as an effective and sensitive means to evaluate the safety profile of new therapeutic targets *in vivo*.

## Introduction

Statins are among the most widely prescribed medications with proven records in decreasing the cardiovascular morbidity and mortality of patients with or without coronary artery diseases; the number of patients indicated to receive long-term statins continually increases [1], [2]. Statins serve the above role through effectively decreasing the level of low-density lipoprotein cholesterol by inhibiting competitively 3-hydroxy-3-methylglutaryl coenzyme A (HMG-CoA) reductase, an enzyme that catalyzes the endogenous synthesis of cholesterol in liver [3]. Although statins are well tolerated by most patients, they can produce myopathy in some patients with varied clinical symptoms ranging from mild myalgia or muscle weakness, to fatal rhabdomyolysis [4], [5]. The deleterious muscle manifestations of statins have received considerable attention, especially after a global withdrawal of cerivastatin in 2001 that claimed about 100 deaths associated with rhabdomyolysis [6]. Despite extensive effort, a controversy about the pathogenesis of statin-induced myopathy remains largely unresolved [7], [8], [9]. Most clinical trials associate the myotoxic effect of statins with a circulating level of creatine phosphokinase (CPK) greater than five to ten times the normal value, but this biomarker does not invariably correlate with clinical symptoms of myopathy because an elevated CPK value can be associated with several diseases; the clinical symptom of myopathy has been difficult to evaluate and can be subjective from patient to patient [10], [11]. Because the enzyme is unstable, whereby its circulating level in blood can be apparently lost before blood sampling, the use of CPK as a reliable biomarker of muscle disease is questioned [12], [13]. Identification of a reliable and specific index that can assess objectively the symptoms or the severity of myopathy is important for an exploration of the pathogenesis and for the development of effective therapies against the disease.

Second-harmonic generation (SHG) is a nonlinear optical process that involves interaction of light with polarizable materials lacking centrosymmetry [14], [15]. It is intrinsic and thus allows imaging of intact tissues or living organisms without labeling; nonlinear excitation enables optical sectioning, and allows use of near infrared excitation to minimize autofluorescence background; photochemical damage of samples is minimized as SHG is a non-resonant process that involves no absorption [16], [17], [18], [19], [20], [21]. SHG imaging has been employed to observe structural proteins such as tubulins in the microtubules of cells, and collagens in tendons, cartridges, dermis and fish scales [22], [23], [24], [25], [26], [27], and thick myosin filaments in myocytes [28], [29], [30], [31], [32].

Fundamental or applied clinical research inevitably involves employment of animals at some stage. The zebrafish (*Danio rerio*) is a genetically accessible organism that possesses a muscular system rapidly developing relative to higher vertebrates such as rodents; the muscle proteins, structure, function and development of zebrafish exhibit extensive homologies with that of humans; its muscular development is visible or readily accessible *in vivo* at all stages [33], [34]. These unique properties render the zebrafish an exceptionally attractive model to assess muscular diseases and the myotoxicity of drugs *in vivo* with optical microscopy [35], [36], [37], [38], [39], [40]. Despite these advantages, little investigation of myotoxicity induced by statin using the zebrafish has occurred. Histological assessment performed in these tests was limited to only specimens that had undergone extensive preparation including fixation, sectioning and staining. The results of structural examination were derived mostly from gross morphological alteration as microscopes with poor spatial resolution were employed, thus missing microstructural information [11], [41], [42]. As sample preparation might distort the fine structure of samples, the results based on the structural modification observed from histological specimens should be interpreted with caution [43]. No quantitative assessment of statin-induced myopathy *in vivo*, especially on the microstructural modification of sarcomeres, is reported.

Here we describe the use of a SHG microscope to assess the myotoxicity of statins in zebrafish. In contrast to conventional histological imaging, observation of the muscle fibers *in vivo* eliminates concern about undesirable structural distortion caused by sample preparation. Analysis of SHG images at high resolution further allows an accurate measurement of the length of sarcomeres, the fundamental contractile unit of myocytes. We demonstrate that statin caused a significant shortening of the sarcomeres of zebrafish muscles while the morphological integrity of the myocyte remained largely intact. This microstructural abnormality is attributed to demolition of the thick myosin filament in sarcomeres, and is associated with the biosynthetic pathway of cholesterol or, specifically, the impaired production of mevalonate by statins. This sarcomeric microstructural disorder exhibited a strong correlation with the dosage and the duration of treatment, a result that indicates a possibility to assess the severity or the symptoms of muscle injury based on the altered length of the sarcomere. In contrast to a conventional assessment of muscle injury using clinical biomarkers in blood, such as creatine kinase that is released from only disrupted myocytes, the ability to determine subtle but unambiguous microstructural modification of sarcomeres allows a diagnosis of muscle injury before an onset of conventional clinical symptoms. In light of the increasing prevalence of the incidence of skeletal muscle injury caused by new therapies, our approach consolidates the combined use of zebrafish and SHG imaging as an effective, rapid and sensitive means to evaluate the safety profile of new therapies on vertebrates *in vivo*.

## Results

### SHG Imaging Enables Microstructural Analysis of Sarcomeres *In Vivo* and Observation of Myofibrillogenesis in Zebrafish

**Figure 1:**
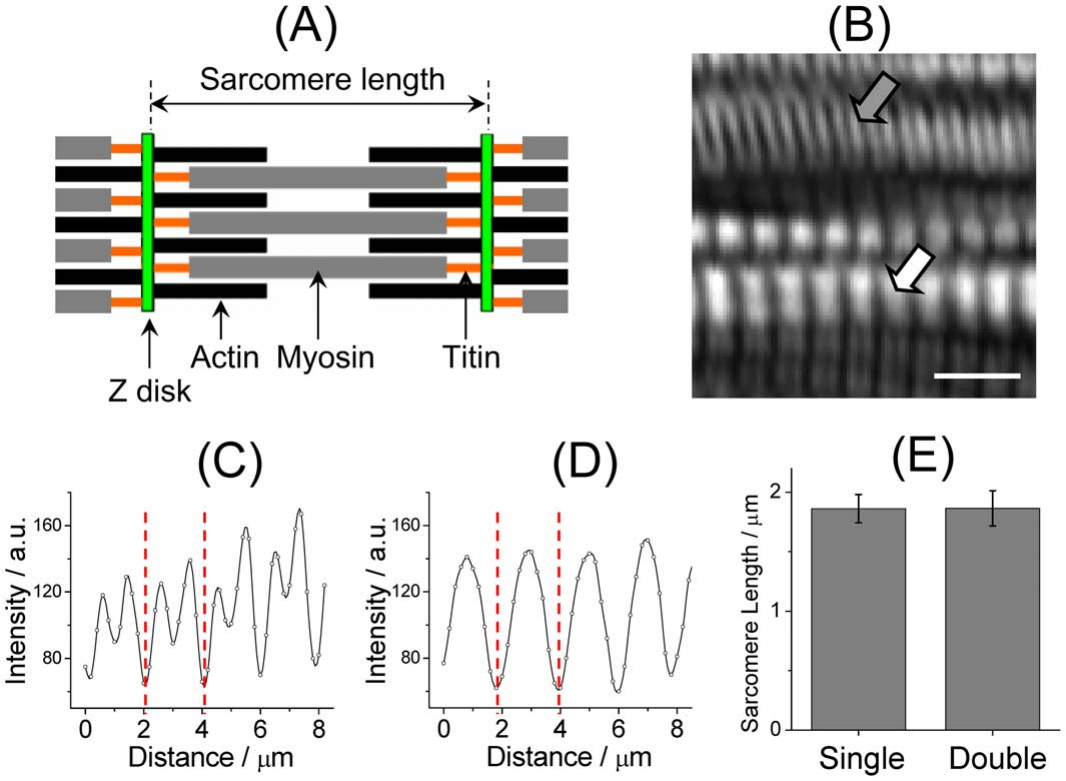
Sarcomeric length determined with SHG imaging. (A) A cartoon illustration of a sarcomere and the architectural arrangement of its major constituent filaments. The length of a sarcomere is defined as the separation between two *z*-disks. (B) A high-resolution SHG image of zebrafish muscles. The image exhibits two characteristic patterns that are denoted as double (gray arrow) and single bands (white arrow), respectively. Scale bar: 5 µm. (C, D) Representative cross-sectional plots of a double band (C) and a single band (D). The length of the sarcomere was determined from the distance between the two dashed lines as shown in the two cross-sectional plots. (E) Comparison of the averaged length of the sarcomere determined from the two sarcomeric patterns. The statistics were calculated based on 12 images obtained from somites near the head (fifth to eighth somite) of three 72-hpf zebrafish.

The myocyte is composed of bundles of myofibrils that contain myofilaments. The thick myosin myofilaments, thin actin myofilaments and titin myofilaments assemble into specific repeating organizations termed sarcomeres that represent the basic contractile unit of the myocyte. **Figure 1A** is a cartoon illustration of a sarcomere. As indicated in the cartoon, the length of the sarcomere is defined as the separation between two *z*-disks. According to the sliding-filament theory of muscle contraction, the thin and thick filaments slide across each other during contraction of the sarcomere, resulting in an increased overlap of the two filaments and thus a shortened sarcomere. **Figure 1B** is a representative SHG image of skeletal muscles measured on a living zebrafish larva (3 dpf). The apparatus of the SHG microscope has been described elsewhere [21], [44]. The regions producing intense SHG signals correspond to the location of thick myosin filaments that arrange in equally spaced arrays in sarcomeres as illustrated in **Figure 1A** [28]. Consistent with the literature [30], two distinct patterns in the image are denoted as *double band* (gray arrow) and *single band* (white arrow), respectively, in conformity with convention. **Figures 1C** and **1D** shows cross-sectional plots of the double and single bands, respectively, in which the two dashed lines correspond to the location of *z*-disks; their separation defines the length of the sarcomere.

Although the origin of the two characteristic patterns exhibited in the SHG image of skeletal muscles has been systematically explored [30], whether the double and single bands shown in the SHG image of zebrafish muscles convey identical sarcomere lengths remains uncertain. To address this question, we performed a systematic analysis of the length of the sarcomere determined from both single and double bands. As shown in **Figure 1E**, the sarcomeres of zebrafish muscles determined from either pattern have indistinguishable lengths (1.86±0.15 µm vs 1.86±0.12 µm, *P* = 0.970). Our reported length is accordingly an average determined from randomly selected regions of the image regardless of either specific pattern unless explicitly specified.

**Figure 2:**
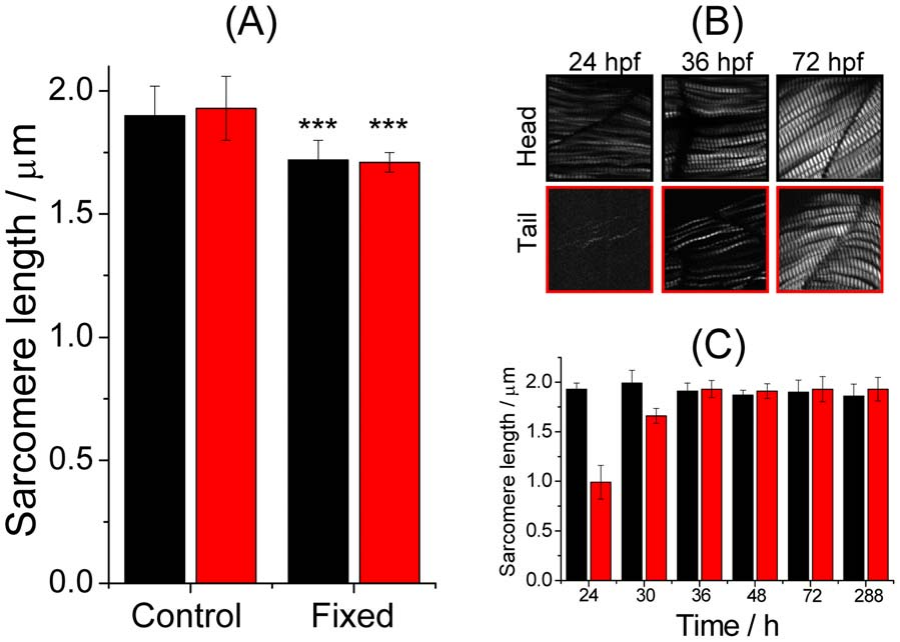
Sarcomeric length of living and fixed zebrafish, and developing larva at different stages. (A) Comparison of length of sarcomeres determined from zebrafish living and fixed with paraformaldehyde. The SHG images used to measure the lengths were measured on somites near the head (black, somites 5–8) and the tail (red, somites 21–24). The statistics were calculated based on 21 images obtained from three 72-hpf zebrafish. (B) Representative SHG images of zebrafish measured at three developmental stages (24, 36 and 72 hpf). The images were measured on regions near the head (somites 5∼8) and the tail (somites 21–24). (C) Growth of the sarcomere from 1 to 12 dpf. The SHG images used to evaluate the length were measured on somites near the head (black, somites 5∼8) and the tail (red, somites 21–24). The statistics were calculated based on 21 images obtained from three 72-hpf zebrafish.

Conventional microstructural analysis of biological tissues is commonly achieved through histological examination with an optical or electron microscope. Distortion of the specimens due to sample preparation is almost inevitable, and might hinder a quantitative structural analysis of tissues. In contrast, observation of living zebrafish with a SHG microscope allows a microstructural analysis of muscle sarcomeres free of distortion. We compared the length of the sarcomeres of living zebrafish with that of zebrafish fixed with paraformaldehyde, a procedure commonly employed in conventional histological analysis. As clearly shown in **Figure 2A**, the sarcomere of the fixed zebrafish is consistently shorter than that of living fish (head, 1.72±0.08 µm vs 1.90±0.12 µm, *P* = 4.81×10^−7^; tail, 1.71±0.04 μm vs 1.93±0.13 µm, *P* = 4.07×10^−8^). This result confirms that fixation of the specimen distorted the microstructure of sarcomeres, and specifically, yielded an erroneously decreased length of the sarcomere.

After demonstrating that a SHG microscope enabled measurement of the length of the sarcomere of zebrafish with superior accuracy through eliminating concerns about structural distortion due to sample preparation, we employed SHG imaging to examine living zebrafish at various developmental stages. **Figure 2B** displays representative SHG images of living zebrafish measured at three representative timings (24, 36 and 72 hpf). Comprehensive analysis of the length of the sarcomere in somites near the head and the length near the tail at multiple developmental stages are shown in **Figure 2C**. As shown there, the sarcomere in somites near the head developed to its full length (∼1.9 µm) as early as 24 hpf. In contrast, the development of sarcomeres in somites near the tail was retarded relative to that near the head; the length of the sarcomere increased from 0.99±0.17 µm at 24 hpf, to 1.66±0.08 µm at 30 hpf, and finally to its full length, 1.93±0.09 µm, at 36 hpf. In subsequent experiments, the length of the sarcomere determined from untreated zebrafish at various stages served also as the control for comparison with the length determined from treated zebrafish.

### Statin Causes a Grossly Altered Morphology and Microstructural Disorder of Sarcomeres of Zebrafish Muscles

**Figure 3:**
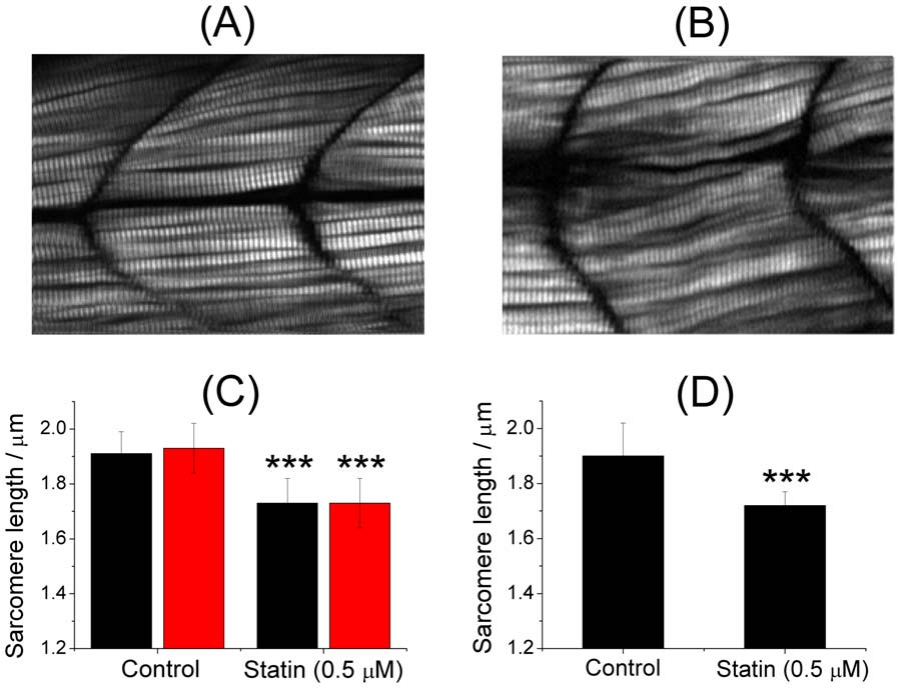
Structural modification of zebrafish muscles induced on treatment with statin. (A) A representative SHG image of the control (an untreated zebrafish larva, 72 hpf). (B) A representative SHG image of a zebrafish larva (72 hpf) subject to treatment with statin (50 µM) for 12 h. Image size: 140 µm (w)×100 µm (h). (C) Effect of statin treatment on zebrafish with underdeveloped sarcomeres. The zebrafish was subject to a treatment of statin (0.5 µM) at 24 hpf for 12 h, and imaged at 36 hpf (black: somites 5∼8; red: somites 21–24). (D) Effect of statin treatment on zebrafish with fully developed sarcomeres. The zebrafish was subject to a treatment of the same dosage at 72 hpf for 12 h, and the image was taken at 84 hpf (head, somites 5∼8).

Representative SHG images of the control (untreated zebrafish) and the treated zebrafish (50 µM) are displayed in **Figures 3A** and **3B**, respectively. The two images exhibit subtle but clearly discernible morphological variation; specifically, the zebrafish treated with statin developed with misorientation, displacement, skewing, or contortion of the muscle fibers.

The SHG images shown in **Figure 3A** and **3B** demonstrate that the treatment of zebrafish larvae with statin induced grossly morphological modification of the muscles, an observation consistent with that reported from the use of immunoimaging, but the latter poorly resolved images allowed only a qualitative and subjective description, rather than a quantitative and objective assessment of the structural alteration. To assess quantitatively the structural alteration associated with myopathy, particularly the sarcomeric distortion induced by the treatment with statin, we analyzed the length of the sarcomere with high-resolution SHG images.

We first examined underdeveloped zebrafish (24 hpf), and display the result in **Figure 3C**. The treatment of zebrafish larvae with statin (0.5 µM) for 12 h caused a significant shortening of the sarcomere relative to the untreated control. The sarcomeres at somites near the head and those near the tail exhibited a similarly decreased length (head, 1.73±0.09 µm vs 1.91±0.08 µm, *P* = 1×10^−8^; tail, 1.73±0.09 µm vs 1.93±0.13 µm, *P* = 4.08×10^−9^).

As the above experiments were performed on zebrafish larvae (24 hpf) of which the sarcomeres at somites near the tail had not developed to their full length, it is possible that the shortening was due to arrested development of the sarcomeres caused by the treatment. To clarify the cause, we performed complementary measurements on zebrafish (72 hpf) with sarcomeres of fully developed length, and display the result in **Figure 3D**. Similar to the result shown in **Figure 3C**, the treatment of statin yielded also significantly shortened 72-hpf zebrafish larvae (1.72±0.15 µm vs 1.90±0.12 µm, *P* = 7.08×10^−7^) relative to the control. As the treatment yielded a similarly shortened sarcomere of zebrafish of which the sarcomeres had already fully developed at the time of treatment, the mechanism of arrested growth of sarcomeres is excluded as the major cause of the sarcomeric abnormality at least for zebrafish with fully grown sarcomeres.

Two other mechanisms might account for the shortening of the sarcomeres. One mechanism is contraction of the sarcomere induced by drugs. According to the sliding-filament theory of muscle contraction, the sliding of the thin and thick filaments results in an increased overlap of the two, thus shortening the sarcomere during contraction. The other mechanism that might explain the decreased length is demolition of the constituent elements of the sarcomere induced by the drug, for example the thick myosin filament. Further structural clues are required to define the mechanism.

**Figure 4:**
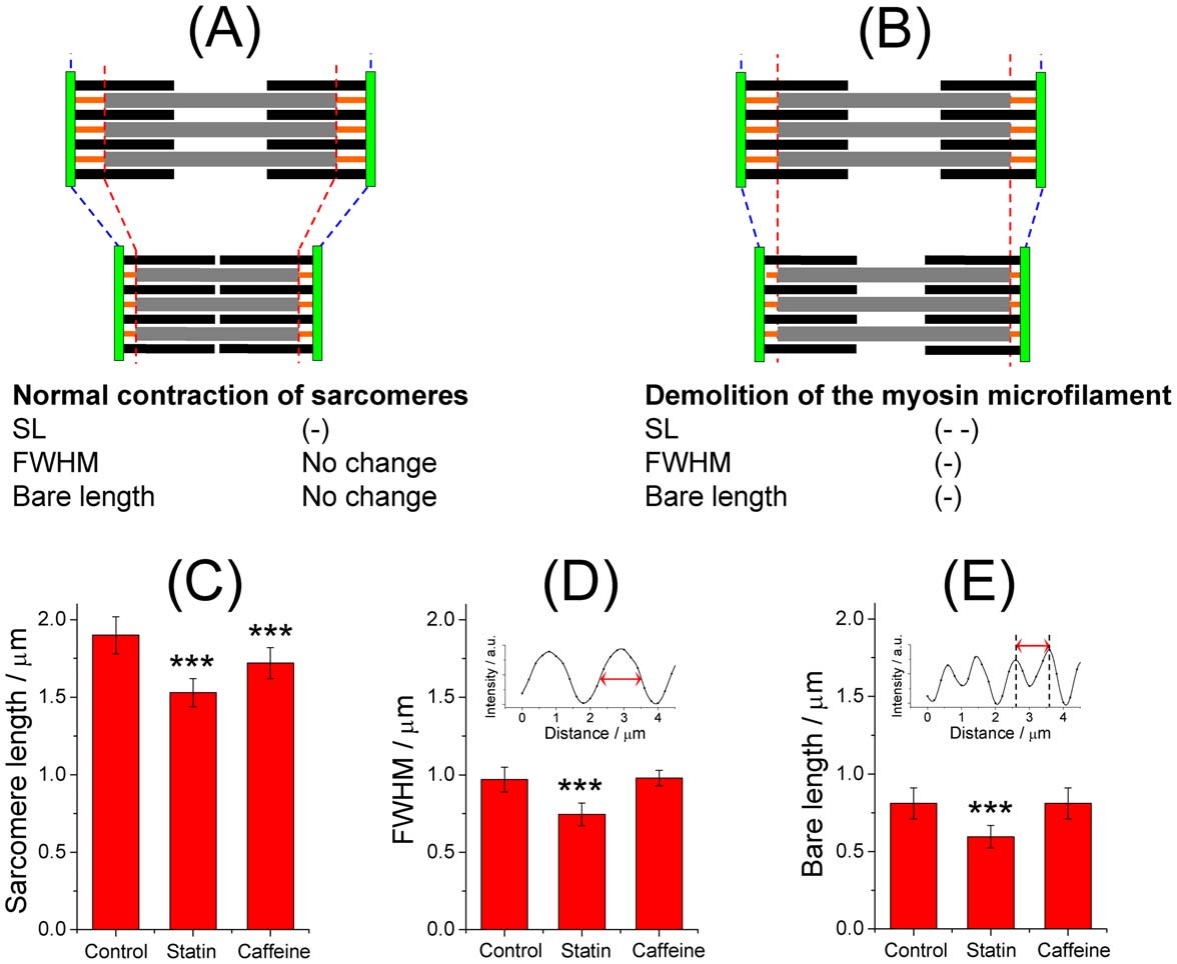
Shortened sarcomeres caused by demolition of the myosin filament. (A) A sarcomere subject to normal contraction. (B) A sarcomere subject to demolition of the myosin filament (B). (C, D, E) Comparison of sarcomeric microstructures of zebrafish subject to no treatment (the control), a treatment of statin (50 µm) at 72 hpf for 120 h, and that of caffeine (8 mM) at 72 hpf for 3 h. All images were obtained at the end of treatments on regions between somites 5∼8. (C) Sarcomere length. The statistics were calculated based on 21 SHG images obtained from three zebrafish. (D) Full width at half maximum (FWHM) of the single band. The statistics were calculated based on 15 SHG images obtained from three zebrafish. (E) Bare length of the double band. The statistics were calculated based on six images obtained from three zebrafish. The FWHM and the bare length were determined on analysis of cross-sectional plots of the single and double bands, respectively, as illustrated in the insets of (D) and (E).

As the SHG signal was produced specifically from the thick myosin filament in the sarcomere, analysis of the separation of the two maxima in the double band (hereafter termed the bare length) and the full width at half maximum (FWHM) of the single band (see also **Figures 1C** and **1D**) would provide structural clues to the integrity of the thick myosin filament. The cartoons displayed in **Figures 4A** and **4B** illustrate the expected modifications of the three microstructural parameters (the length of the sarcomere, FWHM and bare length) for sarcomeres subject to normal contraction or to myosin demolition, respectively. The observation of shortened sarcomeres with intact FWHM and bare lengths would accordingly indicate contracted sarcomeres; in contrast, a concomitant decrease of the three parameters would indicate demolition of the thick myosin filament.

As a test of this hypothesis, we induced persistent contraction of muscle with a treatment of caffeine (8 mM). As shown in **Figure 4C, 4D** and **4E**, the treatment caused a significantly shortened sarcomere (1.72±0.10 µm vs 1.90±0.12 µm, *P* = 3.78×10^−5^) while leaving the FWHM and the bare length intact relative to the untreated control (the FWHM, 0.97±0.08 µm vs 0.98±0.05 µm, *P* = 0.690; the bare length, 0.81±0.10 µm vs 0.81±0.05 µm, *P* = 0.970). These observations are consistent with results expected from the sliding-filament theory of muscle contraction (**Figure 4A**).

Most significantly, analysis of the microstructural parameters for zebrafish treated with statin showed that, in addition to the shortened sarcomere shown before, the FWHM and the bare length also exhibited an appreciable decrease after treatment with statin (sarcomere length, 1.39±0.05 µm vs 1.90±0.12 µm, *P* = 3.15×10^−7^; FWHM, 0.75±0.07 µm vs 0.98±0.05 µm, *P* = 2.52×10^−8^; bare length, 0.60±0.07 µm vs 0.81±0.05 µm, *P* = 2.20×10^−4^). With these results taken together, a detailed analysis of the microstructure of the muscle sarcomere provides unambiguous evidence that the treatment with statin caused demolition of the thick myosin filament, and impaired integrity of the sarcomere, which were manifested as shortened sarcomeres.

### Statin-induced Sarcomeric Disorder Observed with SHG Imaging Is Associated with an Impaired Production of Mevalonate in the Biosynthetic Pathway of Cholesterol

The myotoxicity of statin was suggested to be attributed to the effective inhibition of HMG CoA reductase by statins, resulting in an impaired production of downstream intermediate metabolites such as mevalonate, which is an important precursor of cholesterol in the biosynthetic pathway of cholesterol [45]. Other workers showed that an addition of mevalonate can rescue the morphological damage of muscles caused by statin [11].

**Figure 5:**
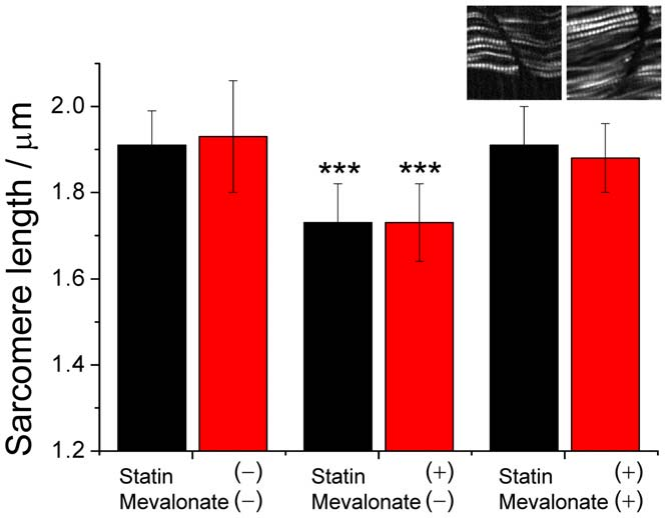
Rescuing effect of mevalonate on the shortened sarcomere induced with statin. Left: the control, untreated zebrafish; middle: zebrafish subject to a treatment of statin (0.5 µM) at 24 hpf for 12 h; right: zebrafish subject to a co-treatment of statin (0.5 µM) and mevalonate (100 µM) at 24 hpf for 12 h. All SHG images used to determine the sarcomere length were measured at 36 hpf on somites near the head (black, somites 5∼8) and the tail (red, somites 21–24). The two images displayed as insets are representative results of co-treatment measured near the head (left) and the tail (right), respectively. Image size: 50×50 µm. The statistics were calculated based on 21 images obtained from three zebrafish.

We have shown that the treatment of statin to zebrafish larval resulted in a significantly shortened sarcomere. To provide unambiguous mechanistic evidence that the microstructural alteration of sarcomeres observed with SHG imaging in living zebrafish is associated also with the biosynthetic pathway of cholesterol, and, specifically, with an impaired production of mevalonate rather than a nonspecific effect, we performed rescuing experiments by co-treating zebrafish (24 hpf) with mevalonate (100 µM) and statin (0.5 µM) for 12 h. The result displayed in **Figure 5** clearly shows that this co-treatment completely prevented the shortening of sarcomere caused by the treatment with statin alone. This result confirms that the observed microstructural alterations of sarcomeres are due also to the inhibition of HMG CoA reductase or the biosynthetic pathway of cholesterol with statins, and, specifically, through an impaired production of mevalonate, rather than a nonspecific effect.

### The Severity of Statin-induced Myopathy Is Correlated with Altered Length of Sarcomere

To demonstrate that SHG images of muscle sarcomeres enables assessment of the severity of myopathy induced by statin, we induced myopathy of varied severity on treating zebrafish with statin of varied dosage and duration, and evaluated whether the altered length of the sarcomere exhibited a correlation with dosage and duration of treatment.

**Figure 6:**
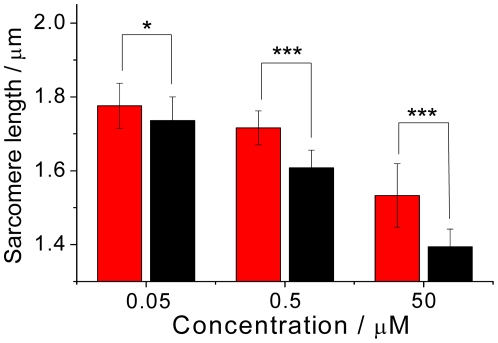
Dependence of the sarcomeric length on the dosage and the duration of treatment. The zebrafish was treated with concentrations 0.05, 0.5 and 50 µM at 72 hpf for either 12 h (red) or 120 h (black). The images were obtained at the end of the treatments on regions between somites 5∼8. The statistics were calculated based on 21 images obtained from three zebrafish.

We first assessed the dosage dependence on treating zebrafish larvae (72 hpf) with statin of three dosages (0.05, 0.5 and 50 µM) while maintaining a constant duration (12 h) of treatment. The results, summarized in **Figure 6**, show that the shortening of sarcomeres exhibited a strong dependence on dosages: the length decreased to 1.78, 1.72 and 1.53 µm from 1.90 µm (untreated control measured at 84 hpf), or 6%, 9% and 21% decrease relative to the control, for dosage 0.05, 0.5 and 50 µM, respectively. The strong correlation of the shortening of sarcomere with dosage indicates that a microstructural analysis of muscle sarcomeres with SHG images allows a quantitative assessment of the severity of myopathy induced by statin.

To consolidate this point, we evaluated the dependence of duration on treating the zebrafish for a duration 120 h for each designated dosage. The results, incorporated in the same figure, show that the sarcomeres exhibited a consistently greater shortening with duration of treatment increasing from 12 h to 120 h for each tested dosage. The prolonged treatment (120 h) caused a shortening of the sarcomere increased by 8%, 15% and 27% relative to treatment for 12 h and dosages 0.05, 0.5 and 50 µM, respectively. This result demonstrates the capability of SHG microstructural analysis of muscle sarcomeres to assess the severity of myopathy induced by statin.

## Discussion

With a SHG microscope, we have determined the length of sarcomere of zebrafish larvae *in vivo* at various developmental stages. Our results, consistent with those reported in the literature [46], indicate that one can observe the myofibrillogenesis of zebrafish *in vivo* without employing labeling reagents as required for immunoimaging. We show also that the length of sarcomeres determined from zebrafish fixed with paraformaldehyde consistently exhibited values erroneously smaller than those determined from living zebrafish. This result demonstrates that SHG images of objects *in vivo* can provide an accurate measurement of the length of growing sarcomeres while eliminating concern about possible structural distortion caused by sample preparation.

The treatment with statin caused demolition of the myosin filament in zebrafish larvae caused significantly shortened sarcomeres of skeletal muscles. With rescue experiments, we provide mechanistic evidence that the microstructural abnormality observed with SHG imaging is associated with an inhibited biosynthetic pathway or an impaired production of mevalonate caused by statin. Such an association of shortened sarcomeres with deleterious muscle manifestations induced by statin is unreported. As the muscle proteins, structure, function and development of zebrafish exhibit extensive homologies with those of humans [33], [34], [38], [47], [48], this finding has clinical significance. As the myosin filament a constituent filament in the sarcomere that is the fundamental contractile unit of muscle fibers, the demolition of the myosin filament might result in impaired muscular functions that translate into clinical symptoms of myopathy.

The severity of the clinical symptoms of myopathy has been shown to depend on the dosage and the duration of treatment with statin [10]. This work shows that the microstructural distortion of sarcomeres, specifically the altered length of sarcomeres, exhibited also a strong correlation with the dosage and the duration of the treatment. This finding might allow an assessment of the severity of myopathy through microstructural analysis of sarcomeres with a SHG microscope. Relative to clinical symptoms of myopathy that have been difficult to evaluate and can be subjective from patient to patient, a microstructural analysis of the sarcomere with SHG might serve as a reliable and objective index for assessment of the severity of myopathy. In light of the increasing prevalence in the incidence of muscle injuries caused by new therapies, SHG imaging combined with the zebrafish models of varied muscular diseases can be particularly useful for an exploration of the pathogenesis of muscle diseases and for the development of effective therapies against these diseases.

In a clinical setting, myopathy is generally diagnosed according to an elevated circulating level of creatine phosphokinase (CPK) that is released from disrupted myocytes, but this enzyme is unstable such that its circulating level in blood can be apparently lost before blood sampling. The use of CPK as a reliable biomarker of muscle diseases has consequently been questioned [12], [13]. Further, this biomarker does not invariably correlate with clinical symptoms of myopathy because an elevated CPK value can be associated with various diseases. A determination of the subtle sarcomeric change associated with myopathy might allow a diagnosis of myopathy before an occurrence of symptoms such as elevated CPK levels.

## Materials and Methods

The Animal Investigation Committee of National Chiao Tung University approved this work (Permit Number: 0099000).

### Reagents

Paraformaldehyde, tricane, mevalonate and lavastatin (Sigma Aldrich) and caffeine (Alfa Aesar) were obtained from the indicated sources.

### Animals

Breeding colonies of AB strain zebrafish (*Danio rerio*) were purchased (Taikong Co., Taiwan) and were kept in small aquaria according to protocols described in the literature [49]. In brief, they were kept in water at 28°C and subject to cycles with light for 14 h and darkness for 10 h. Embryos were generated by natural pair-wise mating. Spawning occurred when the light was just turned on and eggs were collected 30 min afterwards. The collected eggs were further incubated in a flask until subsequent experiments. Zebrafish were staged by hours post fertilization (hpf) and days post fertilization (dpf). At 1 dpf, eggs were examined under a stereo-microscope. Dead or unfertilized eggs were removed.

### Treatment of Zebrafish

A solution of statin or caffeine or mevalonate was prepared on dissolving the reagent in water from the fish tank. The zebrafish were treated on adding the solution to the flask that contained zebrafish at a designed time and for a designed duration, according to experiments. Untreated zebrafish served as the control. Before imaging, the zebrafish were anesthetized with tricane (150 mg/L).

### Apparatus

The apparatus was modified from our home-built multiphoton-imaging system reported elsewhere [21], [44]. A pulsed near-infrared laser (1064 nm, 7 ps, 76 MHz; PicoTran, High-Q Laser, Austria) served for excitation. The pulse train of the laser was directed to an inverted optical microscope (Eclipse Ti, Nikon, Japan) and focused onto the sample with a water immersion objective (CFI Plan Apo 60X, N.A. 1.2, Nikon, Japan). To eliminate the polarization dependence of the SHG signal, the laser light was converted to circularly polarized before entering the microscope. The forward propagating signal was collected with a microscope condenser (N.A. 0.52), spectrally filtered with optical filters, and detected with a thermoelectrically cooled photomultiplier sensor (H-7422-40, Hamamatsu, Japan). Generation of the second-harmonic signal was confirmed with spectral analysis of the emission that exhibits a single spectral line at 532 nm. To increase the ratio of signal to noise, a lock-in amplifier was employed; the SHG images were constructed on recording the demodulated signal in a manner point by point while raster-scanning the sample with respect to the fixed laser focus using a three-axis scanning stage (P-563.3CD, Physik Instruments, Germany) as described before [21], [44]. The laser power, measured at the focus, was typically between 40 and 60 mW. The temporal duration at each pixel was 10 ms. Sample scan, signal collection and image construction were implemented with computer codes (LabView, National Instruments, USA).

### Statistical Methods

The means of two groups were compared with the two-tailed Student's *t* test. The levels of statistical significance were set at *P*<0.05, *P*<0.01, and *P*<0.001, respectively.

### Conclusions

We found that statin caused significantly shortened sarcomeres and the sarcomeric disorder is associated with the biosynthetic pathway of cholesterol impaired by statin. The result indicates the possibility to assess the severity of myopathy and holds a promise for an early diagnosis of myopathy with SHG microstructural analysis of sarcomeres. This approach is extensible to evaluate the safety profile of new therapeutic targets.
